# Supplementary material for: Determinants of knowledge, attitude and self-efficacy towards complementary feeding among rural mothers: Baseline data of a cluster-randomized control trial in South West Ethiopia
Source: PLoS One. 2023 Nov 28;18(11):e0293267. doi: 10.1371/journal.pone.0293267 (PMC10683984; doi:10.1371/journal.pone.0293267)
Supplement: S1 Checklist — (DOCX) [file pone.0293267.s001.docx]

STROBE Statement—checklist of items that should be included in reports of observational studies

|  | Item No. | Recommendation | Page  No. | Relevant text from manuscript |
| --- | --- | --- | --- | --- |
| **Title and abstract** | 1 | (*a*) Indicate the study’s design with a commonly used term in the title or the abstract | 2 | Community based cross-sectional study |
|  |  | (*b*) Provide in the abstract an informative and balanced summary of what was done and what was found | 2 | Overall, half of the mothers had high knowledge. Whereas maternal attitude and self-efficacy towards CF were low |
| Introduction | | | |  |
| Background/rationale | 2 | Explain the scientific background and rationale for the investigation being reported | 3 | Complementary feeding (CF) which is starting from the age of six months with continued breastfeeding up to two years of age or beyond and it is way to reduce child malnutrition |
| Objectives | 3 | State specific objectives, including any prespecified hypotheses | 3 | The main aim of this study is to assess determinants of complementary feeding knowledge, attitude, self-efficacy among rural mothers in Maji Woreda, South West region. |
| Methods | | | |  |
| Study design | 4 | Present key elements of study design early in the paper | 4 | Community based cross-sectional study design was employed as baseline study for a cluster-randomized control trial |
| Setting | 5 | Describe the setting, locations, and relevant dates, including periods of recruitment, exposure, follow-up, and data collection | 4 | This study was carried out in Maji woreda, West Omo zone, Southwest Ethiopia. The data was collected from March 1 to April 3, 2022. |
| Participants | 6 | (*a*) *Cohort study*—Give the eligibility criteria, and the sources and methods of selection of participants. Describe methods of follow-up  *Case-control study*—Give the eligibility criteria, and the sources and methods of case ascertainment and control selection. Give the rationale for the choice of cases and controls  *Cross-sectional study*—Give the eligibility criteria, and the sources and methods of selection of participants | 4-5 | Mothers with infants and young children aged 0-24 months in randomly selected small administrative units were included in the study.  We recruited 516 mothers having an infant and young child using multi-stage sampling techniques. |
|  |  | (*b*) *Cohort study*—For matched studies, give matching criteria and number of exposed and unexposed  *Case-control study*—For matched studies, give matching criteria and the number of controls per case |  |  |
| Variables | 7 | Clearly define all outcomes, exposures, predictors, potential confounders, and effect modifiers. Give diagnostic criteria, if applicable | 5 | Complementary feeding knowledge, attitude and self-efficacy were assessed as an outcome variable. The socio-demographic variables and gynecology-obstetric history were also collected to identify as predictors variables. conducted in different settings |
| Data sources/ measurement | 8* | For each variable of interest, give sources of data and details of methods of assessment (measurement). Describe comparability of assessment methods if there is more than one group | *5* | *A standardized pretested structured interviewer administered questionnaire was employed for the data collection. The adapted questionnaire was from WHO IYCF indicator parameters [17]* |
| Bias | 9 | Describe any efforts to address potential sources of bias | 6 | Pretesting of the questionnaire was done on 5% in the Bench-Sheko zone (other than the study area).  Models were evaluated for potential multi-collinearity using the variance inflation factor, with values less than 10 considered acceptable. |
| Study size | 10 | Explain how the study size was arrived at 4 |  | The sample size was calculated using statcalc (STATA software version-14) with the following assumptions: to detect an increase in appropriate feeding from 7% to 14% [15], with 95% CIs and 80% power, assuming an intra class correlation coefficient of 0.03 [16]. |

Continued on next page

| Quantitative variables | 11 | Explain how quantitative variables were handled in the analyses. If applicable, describe which groupings were chosen and why | 6 | we fitted multiple linear regression models assessing the predictors of complementary feeding knowledge, attitude, and self-efficacy. The scores for the three predictors were standardized based on the distribution of the data, and the results are expressed as regression coefficients with 95% confidence intervals (95%CIs). |
| --- | --- | --- | --- | --- |
| Statistical methods | 12 | (*a*) Describe all statistical methods, including those used to control for confounding | 6 | multiple linear regression models assessing the predictors of complementary feeding knowledge, attitude, and self-efficacy. The scores for the three predictors were standardized based on the distribution of the data, and the results are expressed as regression coefficients with 95% confidence intervals (95%CIs). |
|  |  | (*b*) Describe any methods used to examine subgroups and interactions | 6 | Models were evaluated for potential multi-collinearity using the variance inflation factor, with values less than 10 considered acceptable. Model goodness of fit was assessed using adjusted R2 values. |
|  |  | (*c*) Explain how missing data were addressed | 6 | Missing data were treated |
|  |  | (*d*) *Cohort study*—If applicable, explain how loss to follow-up was addressed  *Case-control study*—If applicable, explain how matching of cases and controls was addressed  *Cross-sectional study*—If applicable, describe analytical methods taking account of sampling strategy | N/A |  |
|  |  | (*e*) Describe any sensitivity analyses | N/A |  |
| Results | | | | |
| Participants | 13* | (a) Report numbers of individuals at each stage of study—eg numbers potentially eligible, examined for eligibility, confirmed eligible, included in the study, completing follow-up, and analysed | 7-8 | mothers participated in the study |
|  |  | (b) Give reasons for non-participation at each stage N/A |  |  |
|  |  | (c) Consider use of a flow diagram N/A |  |  |
| Descriptive data | 14* | (a) Give characteristics of study participants (eg demographic, clinical, social) and information on exposures and potential confounders | 7-8 | socio-demographic characteristics of the participants |
|  |  | (b) Indicate number of participants with missing data for each variable of interest | 7-8 | The total numbers of the participant in the study were 516. |
|  |  | (c) *Cohort study*—Summarise follow-up time (eg, average and total amount) |  |  |
| Outcome data | 15* | *Cohort study*—Report numbers of outcome events or summary measures over time |  |  |
|  |  | *Case-control study—*Report numbers in each exposure category, or summary measures of exposure |  |  |
|  |  | *Cross-sectional study—*Report numbers of outcome events or summary measures | *9-15* | *Mothers’ knowledge about complementary feeding is reported in table 2. Overall, maternal knowledge score was (4.02 ±0.79, M± SD).* |
| Main results | 16 | (*a*) Give unadjusted estimates and, if applicable, confounder-adjusted estimates and their precision (eg, 95% confidence interval). Make clear which confounders were adjusted for and why they were included | N/A |  |
|  |  | (*b*) Report category boundaries when continuous variables were categorized | 7 | Monthly income of the household (ETB) |
|  |  | (*c*) If relevant, consider translating estimates of relative risk into absolute risk for a meaningful time period | N/A |  |

Continued on next page

| Other analyses | 17 | Report other analyses done—eg analyses of subgroups and interactions, and sensitivity analyses | N/A |  |
| --- | --- | --- | --- | --- |
| Discussion | | | | |
| Key results | 18 | Summarise key results with reference to study objectives | 16 -17 | The pertinent finding of this study was more than half of (52.5%) the mothers had high knowledge towards the complementary feeding. |
| Limitations | 19 | Discuss limitations of the study, taking into account sources of potential bias or imprecision. Discuss both direction and magnitude of any potential bias | 18 | The questionnaire used for this study is based on WHO IYCF indicator parameters. A |
| Interpretation | 20 | Give a cautious overall interpretation of results considering objectives, limitations, multiplicity of analyses, results from similar studies, and other relevant evidence | 16-18 | In this study we investigated level and determinants of maternal knowledge, attitude and self-efficacy toward complementary feeding. Even though Ethiopia has attempted to improve complementary feeding, its success has been limited. To the best of our knowledge, there are only a few studies that have used validated questionnaires to assess complementary feeding knowledge and attitude in Ethiopia |
| Generalisability | 21 | Discuss the generalisability (external validity) of the study results 18 |  | These findings imply that nutrition intervention strategies are mandatory particularly to enhance maternal knowledge, attitude and self-efficacy towards optimum complementary feeding. |
| Other information | |  | | |
| Funding | 22 | Give the source of funding and the role of the funders for the present study and, if applicable, for the original study on which the present article is based | N/A |  |

*Give information separately for cases and controls in case-control studies and, if applicable, for exposed and unexposed groups in cohort and cross-sectional studies.

**Note:** An Explanation and Elaboration article discusses each checklist item and gives methodological background and published examples of transparent reporting. The STROBE checklist is best used in conjunction with this article (freely available on the Web sites of PLoS Medicine at http://www.plosmedicine.org/, Annals of Internal Medicine at http://www.annals.org/, and Epidemiology at http://www.epidem.com/). Information on the STROBE Initiative is available at www.strobe-statement.org.
