# Supplementary material for: Determinants of knowledge, attitude and self-efficacy towards complementary feeding among rural mothers: Baseline data of a cluster-randomized control trial in South West Ethiopia
Source: PLoS One. 2023 Nov 28;18(11):e0293267. doi: 10.1371/journal.pone.0293267 (PMC10683984; doi:10.1371/journal.pone.0293267)
Supplement: S1 Table — (DOCX) [file pone.0293267.s002.docx]

**Descriptive statistics of CF knowledge, attitude and self-efficacy**

**Knowledge about CF (n=516)**

| Mothers knew (correct response) | n | % | Mean ±SD |
| --- | --- | --- | --- |
| A child should exclusively breastfed for the first 6 months | 411 | 79.6 | 0.79±0.56 |
| Knew WHO recommended age for complementary feeding initiation is 6 months | 189 | 36.6 | 0.37± 0.21 |
| A breastfed 6–8 months child should to take complementary foods 2–3 times/day | 144 | 27.9 | 0.28±0.19 |
| A breastfed 9–24 months child should take complementary food 4 times/day | 216 | 41.8 | 0.42±0.24 |
| A child 6–24 months of age require a minimum of 4 food groups | 209 | 40.5 | 0.40±0.55 |
| Knew recommended ways to prepare infant’s food | 188 | 36.4 | 0.36±0.11 |
| Consistency of the child's food, according to his/her months of age | 127 | 24.6 | 0.25±0.12 |
| Nutritional or multivitamin supplements should be given | 111 | 21.5 | 0.21±0.10 |
| Foods to prevent the child from developing anemia | 105 | 20.3 | 0.20±0.12 |
| Non-breastfed baby needs extra meal | 383 | 74.2 | 0.74 ±0.31 |
| Classification of knowledge score | **Frequency (%)** | | |
| High | 271(52.5%) | | |
| Low | 245 (47.5%) | | |
| n-frequency of correct responses per item, SD-Standard Deviation | | | |

**Mothers’ attitude towards CF (n=516)**

| Variables | Disagree  n | % | Neutral  n | % | Agree  n | % | Mean ±SD |
| --- | --- | --- | --- | --- | --- | --- | --- |
| Breastfeeding alone is sufficient for a child after 6 months^†^ | 388 | 75.2 | 27 | 5.2 | 101 | 19.6 | 3.01±1.02 |
| Giving complementary foods after 6 months makes a child healthy | 79 | 15.3 | 134 | 26.4 | 303 | 58.7 | 2.81±0.81 |
| It is not good to give child fruits and vegetables^†^ | 144 | 27.9 | 195 | 37.8 | 177 | 34.3 | 2.66±0.55 |
| A child needs animal source foods | 82 | 16.0 | 200 | 38.7 | 234 | 45.3 | 2.13±0.29 |
| Bottle feeding is not good for child’s health | 15 | 2.9 | 183 | 35.4 | 318 | 61.7 | 3.22 ±0.77 |
| Nutritional supplements are affordable and ensure infant has adequate nutrition | 285 | 55.2 | 155 | 30.0 | 76 | 14.8 | 1.58 ±0.25 |
| Giving extra meal is desirable before and after an illness | 66 | 12.8 | 117 | 22.7 | 333 | 64.5 | 2.59±0.63 |
| Complementary feeding is expensive | 21 | 4.1 | 6 | 1.1 | 489 | 94.8 | 4.02 ±1.07 |
| Classification of attitude scores | | | | | | **Frequency (%)** | |
| Favorable | | | | | | 246 (47.7%) | |
| Unfavorable | | | | | | 270 (52.3%) | |
| ^†^ Reverse scored items. | | | | | | | |

**Self-efficacy towards complementary feeding (n=516)**

| Statements | Mean ±SD |
| --- | --- |
| I can continue to breastfed a child to the age of 24 months | 4.13±1.05 |
| I can give complementary foods 2–3 times/day in addition to breastfeeding for 6–8 months child | 3.11±0.83 |
| I can give complementary food 4 times/day in addition to breastfeeding for a child aged 9-24 months | 2.02±0.55 |
| I can give a minimum of 4 food groups required for a child aged 6-24 months | 1.88±0.91 |
| I can give fruits and vegetables to a child to eat. | 1.57 ± 0.44 |
| I can give a child to eat animal source foods | 1.39±0.27 |
| I can give extra meal before and after an illness | 3.02±0.63 |
| I am confident that I can get my child measured if I concerned about his/her growth | 1.42±0.22 |
| I am confident that I can feed my child so he/she gain enough weight | 1.57±0.25 |
| Classification of self-efficacy score | **Frequency (%)** |
| High | 201 (38.9%) |
| Low | 315 (61.1%) |
| Note:5-point Likert scale was used (1=” not at all confident” to 5=” very confident” | |

**-**
