# Supplementary material for: Determinants of knowledge, attitude and self-efficacy towards complementary feeding among rural mothers: Baseline data of a cluster-randomized control trial in South West Ethiopia
Source: PLoS One. 2023 Nov 28;18(11):e0293267. doi: 10.1371/journal.pone.0293267 (PMC10683984; doi:10.1371/journal.pone.0293267)
Supplement: Attachment — Submitted filename: Response to the editor 2.docx [file pone.0293267.s003.docx]

**To: Dr. Jonafer Estoque Miranda
PLOS ONE**
Manuscript number : PONE-D-22-26621
Determinants of knowledge, attitude and self-efficacy toward complementary feeding among rural mothers: Baseline data of a cluster-randomized control trial in South West Ethiopia

We thank the senior editor for their comments and appreciate the opportunity to submit a revised manuscript. The required documents were attached in accordance with the comments and suggestions.

| **Comment** | **Response** | **Page Number** |
| --- | --- | --- |
| Please complete all items on the Clinical Studies Checklist that are relevant for your submission, by following this link: http://journals.plos.org/plosone/s/file?id=dc11/PLOSOne_Clinical_Studies_Checklist.docx (Contact us at plosone@plos.org if you cannot access the document.) There may be overlap between the checklist items and other queries listed below; please address any duplicated queries both in your response email and on the checklist itself. Upload the completed Clinical Studies Checklist as file type “Other” when you re-submit your manuscript. This document is for internal journal use only and will not be published if your article is accepted. The requested information will help us to assess whether your submission complies with PLOS ONE’s policies and adheres to applicable reporting standards. Note that your manuscript may be rejected if you provide incomplete or inadequate responses to the checklist questions and that changing the ‘Section/Category’ of your article does not affect this requirement. | Since this is community based cross-sectional study STROBE checklist along with PLOSOne clinical trial were completed and attached as “other” document. Also the Ethical review letter is attached as “ other” document/ | N/A |
