# Supplementary material for: Determinants of knowledge, attitude and self-efficacy towards complementary feeding among rural mothers: Baseline data of a cluster-randomized control trial in South West Ethiopia
Source: PLoS One. 2023 Nov 28;18(11):e0293267. doi: 10.1371/journal.pone.0293267 (PMC10683984; doi:10.1371/journal.pone.0293267)
Supplement: Attachment — Submitted filename: Response to the editor.docx [file pone.0293267.s004.docx]

**To:** Dr. Richard Ibañez Dilla (**PLoS ONE)**
Manuscript number: PONE-D-22-26621

Manuscript title: “Determinants of knowledge, attitude and self-efficacy toward complementary feeding among rural mothers: Baseline data of a cluster-randomized control trial in South West Ethiopia”

**Comment**

1. Please include a copy of Table 2 which you refer to in your text on page 10.

**Response**

Corrected and the copy of Table 2 included. It was a mislabeling of the table and the number is corrected.

**Comment**

2. Thank you for stating the following financial disclosure:

"The funders had no role in study design, data collection and analysis, decision to publish, or preparation of the manuscript."

**Response**

We declare that we haven’t received any funding from funders. Therefore “The authors received no specific funding for this work.”
