# Supplementary material for: Determinants of knowledge, attitude and self-efficacy towards complementary feeding among rural mothers: Baseline data of a cluster-randomized control trial in South West Ethiopia
Source: PLoS One. 2023 Nov 28;18(11):e0293267. doi: 10.1371/journal.pone.0293267 (PMC10683984; doi:10.1371/journal.pone.0293267)
Supplement: Attachment — Submitted filename: Response to Reviewers CF.docx UraGeb 3.docx [file pone.0293267.s005.docx]

**To:** Dr. Mohan Kumar (Academic editor)

**PLoS ONE**
Manuscript number : PONE-D-22-26621
Manuscript title: “Determinants of knowledge, attitude and self-efficacy toward complementary feeding among rural mothers: Baseline data of a cluster-randomized control trial in South West Ethiopia”

We thank the academic editor and reviewers for their comments and appreciate the opportunity to submit a revised manuscript. The manuscript has been revised in accordance with the comments and suggestions. Overall, the comments are appreciation on the manuscript quality and write-up and really we appreciate for your rigor reviews.

| **Response to editor and reviewer** | **Response** | **Page Number** |
| --- | --- | --- |
| **Response to Reviewer 1** | | |
| **In the abstract:** | | |
| It has not been addressed the following topics:   - How data had been collected?, | -We have accepted the comment and addressed the comment in the method-section of the abstract. | Page 2 |
| - Which are the variables being compared? | -We have accepted the comment and addressed the comment in the method-section of the abstract. | Page 2 |
| - It is not described the two groups that had been compared. | -We have accepted the comment and addressed the comment in the method-section of the abstract. | Page 2 |
| - This sentence needs to be reviewed: "Except for the child’s sex and age, no significant difference was observed between the intervention and control groups in terms of socio-demographic variables (p > 0.05)." | -We have accepted the comment and addressed (revised and rewrote) the sentence accordingly in the result-section of the abstracts. | Page 2 |
| - There is a need to present a confidence interval for the variables that had been identified as statistically significant. | We have accepted the comment and addressed the sentence accordingly in the result-section of the abstracts. | Page 2 |
|  |  |  |
| **Discussion** | | |
| - In the discussion there is a need to present the synthesis of the main results found in this study. | We have accepted the comment and addressed the comment in the discussion part of the manuscript. | Page 13 |
| We thank you the editors and reviewer for the onstructive comments. | | |
